# Supplementary material for: Lessons learned from operationalizing the integration of nutrition-specific and nutrition-sensitive interventions in rural Ethiopia
Source: PLoS One. 2025 Apr 2;20(4):e0290524. doi: 10.1371/journal.pone.0290524 (PMC11964222; doi:10.1371/journal.pone.0290524)
Supplement: S1 File — (DOCX) [file pone.0290524.s001.docx]

**S1 File. Interview guide**

**Tool 1_ Key informant interview with women**

1. How can you recognize that someone is not having enough food?

Probe: What are the signs of undernutrition?

1. What are the reasons why people are undernourished?

Probe: Not getting enough food

Food does not contain enough nutrients

Disease/ill and not eating food

1. What did you hear from HEW/AEW about infant and young child feeding and nutrition-sensitive agriculture recommendations?

Probe: IYCF and cooking demonstration

Home gardening and farm demonstration

Livestock and soil management

1. Did you feel the HEW/AEW were able to assess your situation and your difficulties with child feeding practices and nutrition-sensitive agriculture? How?
2. Did you feel you were able to successfully practice the recommendations that were appropriate and achievable by you? Why or why not
3. Which counselling activities were most helpful/unhelpful and why?
4. What are the barriers that hinders to adopt the recommendations of HEW/AEW on child feeding and nutrition sensitive agriculture practices?

Probe: Cultural and religious sensitivity issues,

Accessibility and availability issues

1. What are your suggestions to improve the IYCF and nutrition sensitive programs in your community?

**Tool 2_ Key informant interview with Extension workers**

1. What are the major causes of undernutrition in your area?

2. What forms of support you provide to the community involved in SURE project?

Please elaborate

3. What factors influence the acceptance of your recommendations of child feeding, cooking food, farm demonstration and gardening in the community?

Probes: Affordability, Availability and Culture

4. What are the benefits gained by the community, you observed due to the SURE project? (Please elaborate)

Probes: Are there any interesting cases reported from the field?

Have you noticed any changes related with the project from your monitoring?

5. What are the challenges that you have faced related with linking health and agriculture sectors in your community?

Probe: What could be done to significantly improve health and agriculture sectors linkage? Please provide specific examples.

6. Do you have any suggestion that can make the project better?

probe: How can this project be made sustainable?

What can be done to improve nutritional status of children and women.
