# Supplementary material for: Lessons learned from operationalizing the integration of nutrition-specific and nutrition-sensitive interventions in rural Ethiopia
Source: PLoS One. 2025 Apr 2;20(4):e0290524. doi: 10.1371/journal.pone.0290524 (PMC11964222; doi:10.1371/journal.pone.0290524)
Supplement: S2 File — (DOCX) [file pone.0290524.s002.docx]

**Checklists for reporting qualitative studies**

| SNo | Items | Guiding questions/Description | Remark |
| --- | --- | --- | --- |
|  | **Domain 1: Research team and reflexivity** | |  |
|  | ***Personal characteristics*** | |  |
|  | Interviewer/facilitator | EZ, TK, BW,MW conducted the interview |  |
|  | Credentials | MSc, PhD, MD/PhD* |  |
|  | Occupation | Researchers and academician |  |
|  | Gender | Both involved |  |
|  | Experience and training | Special training on qualitative data collection |  |
|  | ***Relationship with participant*** |  |  |
|  | Relationship established | Relations established |  |
|  | Participant knowledge of interviewer | The participants know about the interviewer as a project team |  |
|  | Interviewer characteristics | Interest in the research topic considered and reported |  |
|  | **Domain 2: Study Design** |  |  |
|  | ***Theoretical framework*** |  |  |
|  | Methodological orientation and theory | Phenomenology design used |  |
|  | Sampling | Purposive sampling was used |  |
|  | Method of approaches | Face to face data collection was used |  |
|  | Sample size | 28 key informant interviews |  |
|  | Non participation | No missing participant |  |
|  | ***Setting*** |  |  |
|  | Setting of data collection | Data collected at community health post and farmers training centers with optimal seating |  |
|  | Presence of non-participants | non-participants not presented |  |
|  | Description of sample | The interviews are with women health extension workers and agriculture extension workers |  |
|  | ***Data collection*** |  |  |
|  | Interview guide | Pretested interview guide was used |  |
|  | Repeat interview | Repeat interview not conducted |  |
|  | Audio/visual recording | Audio recording was used |  |
|  | Field note | Field notes were used to complement audio records |  |
|  | Duration | KII last 20-55 minutes |  |
|  | Data saturation | Data saturated explained |  |
|  | Transcript returned | No | Due to security reasons after the translation of transcripts were not returned to participants |
|  | **Domain 3: Analysis and finding** |  |  |
|  | ***Data analysis*** |  |  |
|  | Number of data coders | Two data encoders code the data |  |
|  | Description of coding tree | Process of coding described |  |
|  | Derivation of theme | Sequential derivation of theme discussed |  |
|  | Software | Nvivo 12 |  |
|  | Participant checking | No | Due to security reasons after the translation of transcripts were not returned to participants |
|  | ***Reporting*** |  |  |
|  | Quotations presented | Quotations presented to support finding |  |
|  | Data and finding consistent | Yes |  |
|  | Clarity of major themes | Themes are defined and discussed |  |
|  | Clarity of minor themes | Minor themes are defined and discussed |  |
