# Supplementary material for: Lessons learned from operationalizing the integration of nutrition-specific and nutrition-sensitive interventions in rural Ethiopia
Source: PLoS One. 2025 Apr 2;20(4):e0290524. doi: 10.1371/journal.pone.0290524 (PMC11964222; doi:10.1371/journal.pone.0290524)
Supplement: Attachment — Submitted filename: Response to Reviewers.docx [file pone.0290524.s004.docx]

| **Response to reviewers** | |
| --- | --- |
| **Academic editor Comment** | **Authers response** |
| There are grammar and language flaws that needs intense revisions. Go throughout the entire document and make revisions. | Thank you for the recommendations and we have done the grammar and language flows as recommended. |
| Briefly explain how you ensured adequacy of the sample size? | Thank you for the clarification questions by the academic editor. We have addressed them in detail.  Participants were purposively selected based on specific characteristics, particularly their active participation in the implementation of the program, which is central to our research question. Adequacy of the sample was ensured by intentionally selecting a diverse range of participants who could provide rich, varied insights, covering all relevant aspects of the research question. When interviews began to produce redundant information, it indicated that the sample size was adequate, meaning data saturation had been reached, with no new information emerging. Therefore, the adequacy of the sample size was ensured by obtaining maximum information from both implementers' and beneficiaries' perspectives until saturation was achieved, line 192-194 |
| How you ensured the quality of qualitative data in this study? | The data collectors received detailed training on the project's objectives and implementation. To ensure data quality, they underwent intensive training in qualitative research data collection techniques. After the training, a conducive environment was created for them to practice using the topic guides before beginning actual data collection. During the data collection phase, supervisors were assigned to each region to coordinate the interviews and ensure that all procedures were followed to obtain accurate and relevant information, line (185-189) |
| Briefly describe who were the data collectors and supervisors? | The Ethiopian Public Health Institute has a pool of data collectors and supervisors for various research data collection efforts. For our study, data collectors and supervisors were purposively selected from this pool based on specific criteria: previous qualitative data collection experience, relevant educational background, and proficiency in the local language. Additionally, to be included in our team, they were required to have recent experience within the past 12 months. The data collectors had educational backgrounds in nursing, laboratory technology, and health officer. Supervisors held a master's degree in public health or nutrition, line (180-184). |
| **Reviewer Comment** | **Authers response** |
| **Abstract** | |
| Use proper tenses e.g instead of the study aims, it should be the study aimed; A qualitative study has been conducted should read ….. study was conducted | Thank you for the recommendations . We have corrected accordingly. |
| Define briefly what was the large intervention program | Comment accepted and the definition of the large intervention has included, line….. |
| The first two sentences of the result are describing the intervention rather than the findings of the study. | We acknowledge the reviewer’s comments, and we have described the interventions accordingly. These two sentences were result of our study and now we make it clearer. |
| The authors claims that the study was done in 4 agrarian areas but one region was removed due to war. In the description of the intervention can be 4 but this study was done in 3 agrarian areas | Thank you for the comment and we make clear it in the method section. The program intervention was implemented in four regions. However, due to the conflict in the Tigray region, we were unable to collect data from that area for the current article. All the result section is explaining about the three regions(Amhara, Oromia and SNNPR). We stated this issue clearly in the method part. |
| **Background** | |
| Provide citations for statement from line 43 to 45 | Thank you and the citation corrected accordingly, line 50 |
| The following statement should be revised into two sentences ‘Young children and women of reproductive age are particularly vulnerable because of their increased nutrient requirements, and, in case of women, their lower social status, which limits their access to nutrient-rich foods’. | We acknowledge your recommendation and corrected as per the recommendation, line 50-53  We change the statement as follows: Due to their increased nutrient requirements young children are vulnerable to undernutrition. Additionally, women of reproductive age are vulnerable to undernutrition because of their lower social status, which limits their access to nutrient-rich foods. |
| The third paragraph (lanes 68 -80), the authors claim some facts but no citations in the whole paragraph. These claims should be supported with citations | Thank you, comments accepted and now citations are included accordingly, Line (76,78,80 and 84). |
| On line 83, EDHS 2016 was used for prevalence of stunting, underweight and wasting while there is EDHS 2019. Do the authors want to refer before the intervention? | Thank you for the comments and we have enriched our evidence by including the magnitude of under nutrition from 2019 EDHS finding too. The EDHS 2016 was the baseline reference figure used to design the program interventions for measure progress of undernutrition in Ethiopia. |
| I was just curious as to if there had been any quantitative evaluations of the Sustainable Undernutrition Reduction Program in Ethiopia (SURE) program published; if so, it would be preferable to include the results in the background. | Thank you for your interest in enhancing our study with quantitative evidence. We appreciate your valuable points. While the project evaluation has not been completed, the Ethiopian Public Health Institute conducted a study on the program's coverage at the end of the project. Now, we have included the published findings from this program coverage survey in the background section, line (116-118) |
| **Method** | |
| Authors should clearly describe what was/were the component(s) for nutrition-specific intervention and for nutrition-sensitive intervention used under the Sustainable Undernutrition Reduction program in Ethiopia (SURE). Could the authors more clearly identify for the reader which intervention parts are nutrition-specific versus those that are nutrition-sensitive? | Thank you for the comments and now we have included the description of each intervention under each term (Nutrition specific nutrition sensitive interventions)  Examples of Nutrition specific interventions that were included as part of the project were child feeding practices, diet diversity, treatment of child illness.  Nutrition sensitive interventions that were part of the intervention were promoting the production diverse food, WASH and women empowerment, which in turn supports better nutrition outcomes. For instance, promoting home gardening to grow vegetables and fruits, improving access to micronutrient-rich foods, agricultural inputs were among the nutrition sensitive interventions. |
| It is not clear why authors are including control districts in the method section instead of description of the intervention districts | Thank you and we accept the reviewer’s comments and corrected accordingly. Now we have excluded the information about the controls in the method section and we have included description of the intervention districts. |
| What was the justification of selecting one Kebele per district randomly? | Thank you for your questions. The program implementers (extension workers) were centrally trained and believed to have similar understanding of the project implementation. The research team believes that selecting one kebele is sufficient to gather information about the project's implementation. We understand that the performance of each kebele implementer may vary; for instance, one may perform better while another may perform worse. However, we believe that selecting a kebele at random allows us to explore data from both scenarios, as there is an equal probability of choosing a higher- or lower-performing kebele due to the random selection process. |
| Do you believe the findings would have been different if you had chosen the Kebeles with the highest or lowest percentage of maternal or child malnutrition? | To the best of our knowledge, there is no evidence in Ethiopia that shows the stratified prevalence of maternal and child undernutrition at the kebele level. During the design phase of our project, we only had access to district-level prevalence data on undernutrition. Consequently, the matching of controls and districts was based on district-level stunting prevalence and food security status. Since the intervention is community-based and the program implementers share similar backgrounds, we do not anticipate significant differences in results between areas with the highest and lowest percentages of undernutrition. However, the reasons behind the varying prevalence rates are important to consider. For instance, factors such as community awareness, food security status and infection rates in the kebeles could influence the results, leading to variations in the percentages of maternal and child nutrition. |
| The intervention started in 2016 and data collection was conducted in 2021, so the data collection was done after 5 years not 4 years. | Thank you for the questions. The actual program was initiated in October 2016, following the completion of baseline data collection between April and June 2016. After a project preparation period, the program was implemented for 4 years and a few months and concluded in December 2020. Subsequently, we conducted the current study in the first quarter of 2021, with data collection taking place in January 2021. This timeline results in a total program duration of four complete years. |
| How were the 28 Key Informants sampled/reached? According to Table 1 there are 9 Agriculture extension workers, 10 Health extension workers and 9 Mothers. For example, what were the basis or criteria to select 9 mothers purposively in each Kebele? Explanation is needed also how out of 36 districts arrived to those numbers? | The key informants were purposefully selected by assuming the potential source of information to attain the objective of our study as implementers (extension workers) and direct beneficiaries of the interventions (mothers). The mothers were chosen not only because they were project beneficiaries but also because they were active participants in women's group dialogues and influential in mobilizing other mothers for cooking and farming demonstrations. The sample size was determined when data collectors reached a saturation point(no new information is expected from further interview) during the interviews on the subject matter. Our study is part of a large quantitative study design and prior to the baseline survey, 36 intervention districts were randomly selected within four regional strata with the number per strata determined in roughly equal proportions to the 50 intervention districts. To reach the required sample size of the baseline study, 36 districts out of 50 were adequate. The selection of the districts for intervention was done by the ministry of health based on the severity of food security and stunting prevalence. |
| **Result** | |
| Overall comment: the findings are not aligned to the topic of the study. There some themes, not related to the lessons learned from the intervention. E.g Causes of malnutrition …. Does it mean that they came to know the causes after intervention??? | Thank you for the comments. The causes of undernutrition are multidimensional, context-specific and seasonal. We believe it is important to provide background information to our readers about the understanding of participants about what is undernutrition and its causes before sharing their experience on the program intervention, exploring the challenges and opportunities of the program. Some participants may perceive poor leadership and management of services as contributing factors to undernutrition, while others may identify immediate causes like inadequate food or disease. Therefore, we included this information at the beginning of the interviews to gain deeper insights into their understanding of nutrition and its causes. It doesn’t mean they know the causes after intervention, but they share actual magnitude of the problem and causes, which help to explain the existing scenario to our readers within the existing context. |
| What were the perceived positive impact of the intervention?  What were the challenges faced during intervention?  Are there any recommendations proposed by the participants? | Thank you for your comments to reflect on the project impact, challenge and recommendations.  We have learned that support of family members in childcare and low incidence of severe cases of undernutrition were among the positive effects of the project in the community.  The presence of high workload on extension workers, civil unrest, shortage of agricultural inputs, limited training and poor supportive supervision and COVID-19 pandemic were the most frequently reported challenges mentioned by the study participants.  They have suggested to strengthen training, assign appropriate manpower, adequate budget for implementation and close supervisions were recommendations forwarded by the participants. |
| So, it is recommended that, first authors should come up with a Table indicating the themes and sub-themes. This will facilitate accurate analysis and interpretation while preventing muddle and confusion as it is now | Thank you for the comments. The analysis was done by arranging the data into certain themes areas and subthemes. The themes of the analysis have been presented in the method section. |
| **Discussion** | |
| Is paragraph 1, relevant to the topic and objective of the study? | Thank you for the comments and we have corrected accordingly. The discussion part has been now included many inputs based on the reviewer comment. |
| The discussion is not discussing point by point according to the findings. Authors should re-write this section by aligning to the key themes and sub-themes. | Thank you for the suggestions and we have rewritten the discussion part in detail as per the recommendations. |
| The authors should discuss the literature of other similar interventions (such as SUAAHARA, ASTUTE, <https://doi.org/10.1016/j.cdnut.2022.100018>, etc.) and fit their findings into the body of knowledge already available on implementation research for these significant multi-sectoral programs and what this study adds to that body of knowledge. | Thank you, we found the suggested article interesting and included in the discussion part as per the reviewer comments. We have also discussed other articles. |
| **Strengths and limitations.** | |
| • This section is poorly written and seems the inclusion and exclusion criteria | Thank you for the comment and corrected accordingly. Now this section has improved as per the recommendation by the reviewer, line 511-513 |
| • Authors could be more thoughtful with the limitations, both with the participants and the bias from the program implementers | Thank you and corrected accordingly, line514-517 |
| **Conclusion** |  |
| Not well written to reflect the findings. What recommendations can be given to address the challenges to enhance impact and uptake of future programs? | Thank you for the comments and now we have corrected it. We have included as per the recommendations of the reviewer. |
| **Overall comments** • Adjust the typos and grammatical errors throughout the document | Thank you, we have corrected the typo and grammatical errors accordingly. |
