# Supplementary material for: Lessons learned from operationalizing the integration of nutrition-specific and nutrition-sensitive interventions in rural Ethiopia
Source: PLoS One. 2025 Apr 2;20(4):e0290524. doi: 10.1371/journal.pone.0290524 (PMC11964222; doi:10.1371/journal.pone.0290524)
Supplement: Attachment — Submitted filename: Response to Reviewers round_2.docx [file pone.0290524.s005.docx]

| **Response to reviewers** | |
| --- | --- |
| **Reviewer Comment** | **Authers response** |
| **Abstract** | |
| To be more specific and reflecting the topic: the integration of nutrition-specific and nutrition-sensitive interventions should incorporated in the background of the abstract. For instance, “The study aimed to explore the experience of health and agriculture extension workers in integrating nutrition-specific and nutrition-sensitive interventions provided to households……. | Thank you for your suggestion and corrected accordingly |
| **Method** | |
| Again, to reflect the topic authors should clearly outline what was/were the component(s) for nutrition-specific intervention and for nutrition-sensitive intervention used under the Sustainable Undernutrition Reduction program in Ethiopia (SURE). They were indicated in the response of point-by-point but not in the manuscript. | Thank you and now the responses are included in the main manuscript.  Line 135 -139 |
| **Result** | |
| The result section was not adequately addressed as per the comments given before. For example, authors responded, “We have learned that support of family members in childcare and low incidence of severe cases of undernutrition were among the positive effects of the project in the community. The presence of high workload on extension workers, civil unrest, shortage of agricultural inputs, limited training and poor supportive supervision and COVID-19 pandemic were the most frequently reported challenges mentioned by the study participants. They have suggested to strengthen training, assign appropriate man-power, adequate budget for implementation and close supervisions were recommendations forwarded by the participants.” however, these are not reflected in the result. | Thank you for the comments and now the result section has reflected the points mentioned in the first version of comments. |
| Aside from the theme of challenges, the results are not clearly presented. After outlining the socio-demographic characteristics, the authors should explicitly highlight the main themes and sub-themes to enhance reader comprehension. | Thang you for the valuable suggestions and We have made revision in the result section including the thematic areas under the demographic Information. We hope the result is clear and satisfy our reviewer. |
| The theme of "Lessons learned and sustainability of the health and agriculture intersectoral intervention" is mentioned, but there are no clear key lessons provided that could be applied to other settings. | We made revisions on the result section and the key lessons from the project are the knowledge gain in childcaring and reduction of severe undernutrition (wasting) cases can be achieved through joint health and agriculture interventions in rural communities. |
| The data analysis procedure was well described but not aligned with result. | Thank you for the comments and in this version, We tried to align the result with the data analysis. |
| Some of the quotations are quite lengthy, making them difficult to follow. It would be helpful to either shorten them or include additional, more concise quotations for clarity | Thank you for the comments and we have corrected the quotations accordingly. |
| **Discussion** | |
| The discussion is not discussing point by point according to the findings. Authors should re-write this section by aligning to the key themes and sub-themes. | The discussion is now aligned with the result . |
